# Hospital-physician relations: the relative importance of economic, relational and professional attributes to organizational attractiveness

**DOI:** 10.1186/1472-6963-14-232

**Published:** 2014-05-21

**Authors:** Jeroen Trybou, Paul Gemmel, Yves Van Vaerenbergh, Lieven Annemans

**Affiliations:** 1 Department of Public Health, Ghent University, De Pintelaan 185, Gent B-9000, Belgium; 2 Department of Management, Innovation and Entrepreneurship, Center for Service Intelligence, Ghent University, Tweekerkenstraat 2, Gent B-9000, Belgium; 3 Research Center for Human Relations, KU Leuven, Warmoesberg 26, Leuven 1B-000, Belgium; 4 Faculty of Medicine and Pharmacy, Vrije Universiteit Brussel, Pleinlaan 2, Brussels, Elsene B-1050, Belgium

## Abstract

**Background:**

Belgian hospitals face a growing shortage of physicians and increasingly competitive market conditions. In this challenging environment hospitals are struggling to build effective hospital-physician relationships which are considered to be a critical determinant of organizational success.

**Methods:**

Employed physicians of a University hospital were surveyed. Organizational attributes were identified through the literature and two focus groups. Variables were measured using validated questionnaires. Descriptive analyses and linear regression were used to test the model and relative importance analyses were performed.

**Results:**

The selected attributes predict hospital attractiveness significantly (79.3%). The relative importance analysis revealed that hospital attractiveness is most strongly predicted by professional attributes (35.3%) and relational attributes (29.7%). In particular, professional development opportunities (18.8%), hospital prestige (16.5%), organizational support (17.2%) and leader support (9.3%) were found to be most important. Besides these non-economic aspects, the employed physicians indicated pay and financial benefits (7.4%) as a significant predictor of hospital attractiveness. Work-life balance and job security were not significantly related to hospital attractiveness.

**Conclusions:**

This study shows that initiatives aimed at strengthening physicians’ positive perceptions of professional and relational aspects of practicing medicine in hospitals, while assuring satisfactory financial conditions, may offer useful avenues for increasing the level of perceived hospital attractiveness. Overall, hospitals are advised to use a differentiated approach to increase their attractiveness to physicians.

## Background

Worldwide, hospitals face challenging times. Physicians play a central important role in shaping the increasingly competitive environment in which hospitals operate. First, in response to financial pressures, hospitals attempt to realize economies of scale and adopt strategies dedicated to increase the flow of patients into the hospital. The primary strategy has been described as a ‘medical arms race’ in which hospitals compete by increasing their share of physicians who admit patients to the hospital in order to maximize hospital occupancy rates [1]. In this sense hospital competition for patients and market share occurs on the physician level. Second, while hospitals traditionally faced only competition from other hospitals, today’s health care delivery is characterized by the proliferation of physician-owned outpatient facilities that potentially compete with full-service hospitals [2]. Third, in many countries hospitals are confronted with a chronic physician shortage and an exponential increase in the demand of care [3,4]. Since the growth in demand is likely to intensify because of ongoing progress in medical science, emerging new technologies and ageing populations [5], physician retention is a hospital management priority.

In this challenging environment hospitals have been struggling to build effective hospital-physician relationships [6] which have been pointed out as a critical determinant of organizational success [7]. Considering the confluence of these forces, it is not surprising that Hospital-Physician Relationships (HPRs) are an important area of academic research and a key concern of hospital managers and health policy makers. Moreover, given the central important role of physicians in hospital care delivery, it has been shown that HPRs have an impact on quality of provided care [8], hospitals’ financial performance [9] and cost-effectiveness of health care delivery [10].

Previous research has offered a number of important insights into the management of HPRs ranging from a financial view with a focus on alignment of incentives to a non-economic focus which aims at optimizing the working environment of physicians [11]. In this paper we build further on this endeavor by investigating the relative importance of several organizational attributes (economic, relational and professional) to physicians. This study was guided by psychological contract theory and the concept of an attractive organizational image to investigate the relative importance of hospital attributes to physicians and increase insight into the complex hospital-physician relationship. Surprisingly, no previous studies have explored hospital image beliefs of physicians. Yet, such an examination is of practical and theoretical importance.

First, although the concept of an attractive organizational image has received a lot of theoretical attention, relatively few empirical studies have examined this issue. Moreover the available studies have focused primarily on potential applicants’ impressions of organizations as employers in the recruitment process. While these studies have increased insight on the factors driving organizational attractiveness for job seekers [12] we do not yet know what determines attractiveness for those people already working at the organization. Furthermore, despite its importance the content or basis of these impressions has remained virtually unexplored [13]. From a hospital perspective, it should be clear how the image of a hospital determines the attractiveness for physicians to work for that particular organization. In light of the physician shortage [4], the physician fled to ambulatory facilities [2] and increased competition between general hospitals [14], the concept of hospital attractiveness is of major importance.

**Figure 1:**
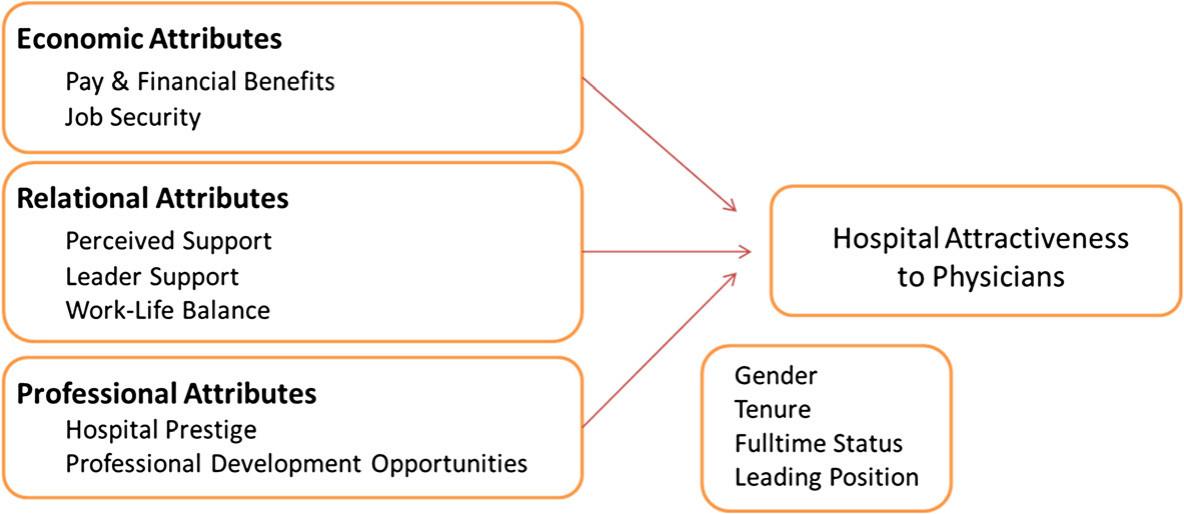
Conceptual framework.

Second, from an academic point of view it might be interesting to know which organizational attributes (economic, relational and professional) are important to professional employees. Moreover, empirical evidence demonstrates that employee-organization dynamics are more complex than has been acknowledged previously [15] and that professional employees like physicians do not adhere to reciprocity principles in a straightforward fashion as originally conceived to be [16]. Although previous research has stressed the importance of economic [17], relational [18] and professional [19] aspects, no previous studies have explored these dimensions of the HPR simultaneously and little is known about their relative importance. Figure 1 provides an overview of the conceptual framework guiding this study.

### Theoretical background and hypotheses

In recent years the concept of an attractive organizational image has received increasing attention within the field of human resource management. In its essence, the organizational image can be described as a mixture of attributes, tangible or intangible, symbolized in a trademark, which can be managed to create value and influence [20]. Since different organizational attributes contribute to this image we argue that this concept is closely related to the concept of the psychological contract. More precisely, the psychological contract consists of individual’s beliefs regarding terms and conditions of the exchange between the individual and his or her organization [21]. It refers to the way the working relationship is interpreted, understood and enacted. Psychological contract theory is considered to be one of the most influential theories to understand organizational behavior. There has been a multitude of studies on the psychological contract between employee and organization over the last 20 years, demonstrating the explanatory power of psychological contract fulfilment and/or breach to a variety of work-related attitudinal and behavioural outcomes [22].

Applied to the HPR, the theoretical concept of the psychological contract enables us to study physicians’ perceptions of specific hospital attributes (the content of the psychological contract) which shape the organizational image. Furthermore we determine to what extent these perceptions (the evaluation of the fulfillment of the contents of the psychological contract) predict organizational attractiveness.

Moreover, it has been shown that physicians do not have exactly the same objectives or motivations as the organization and do not necessarily act in the best interest of the organization [11]. Organizational attractiveness provides a way of accounting for this agency problem associated with employment relationships [23]. In support of this assumption, previous research demonstrated that physicians’ perceptions of their healthcare organization’s image were positively associated with their tendency to engage in cooperative and organizational citizenship behaviors [24]. In this respect, psychological contracts have an important impact on hospitals’ ability to attract, retain and motivate scarce physicians.

Clearly, many aspects determine physician’s perceptions of hospitals attributes thereby shaping the organizational image. One aspect of an organization’s offering will be the financial conditions. Prior research focusing on HPRs has paid a lot of attention to the economic arrangements between hospital and medical staff members [17], an aspect that has dominated previous research [11]. Building on these insights we included two attributes reflecting the economic relationship: physicians’ perceptions of the degree to which he or she is fairly rewarded (pay and financial benefits) and job security.

Hypothesis 1: Hospital economic attributes (pay and financial benefits and job security) are positively related to the perceived attractiveness of the hospital as an employer.

While the economic approach has been widely used to increase insight into the complex issue of hospital-physician relationships, these studies have been criticized because they assume that human motivation is primarily based on self-interest and ignore the fact that economic transactions are embedded in social relationships [25,26]. They fail to recognize that physicians have a more complex set of motives that underlie their behavior [27].

Besides these economic rewards, intrinsic rewards provided by hospitals will fulfill for example socio-emotional needs. As such, the employment experience is made up of a complex array of features [28]. Prior research on HPRs has made a similar distinction between the economic-financial relationship and the relational perspective (non-economic relationship) focusing on the cooperative nature of the day-to-day working relationship [11]. Outside the context of HPRs, considerable research has been conducted on the impact of quality of exchange relationships with the organization (perceived organizational support) and leader (leader-member exchange) on a multitude of work-related attitudes and behaviors. Specifically, perceived organizational support and leader-member exchange has been related to a variety of work-related outcomes such as affective commitment, trust and intention to leave [29] and organizational citizenship behavior [30]. Furthermore, in the past decade there has been increasing interest of organizational researchers in the concept of work-life balance. The business case for work-life balance practices relies on the ability to reduce work-life conflict among employees thereby improving employee attitudes and behaviors within the organization [31]. We build further on this insight by including these three attributes: the perceived degree to which the hospital values and listens to its employees (organizational support), the perceived degree to which the immediate leader can be relied upon and is willing to listen to job-related problems (leader support) and the degree to which the hospital offers good working hours and makes efforts to meet physicians’ expectations of work-life balance (work-life balance).

Hypothesis 2: Hospital relational attributes (perceived support, leader support and work-life balance) are positively related to the perceived attractiveness of the hospital as an employer.

Finally, when considering non-economic aspects of the HPR, it has been recognized that an ideologically pluralistic work setting is present. In hospitals ideologies of professional work bump up against ideologies of the administrative organization in determining the appropriate terms of the physician employment relationship [19,32]. In other words, physicians interact with the hospital both as professional and as employee. Both roles shape HPRs and determine a set of a priori expectations about roles, rights and obligations. Two specific professional expectations were included. First, we included the degree to which the hospital is highly regarded and respected (hospital prestige). Since the organization under study has an academic status which distincts the hospital from (non-academic) general hospitals this could be an important attribute from a professional point of view. Second, the perceived opportunities for training and education (professional development opportunities) were included. More precisely, this refers to the ability of physicians to acquire and improve their professional skills and knowledge.

Hypothesis 3: Hospital professional attributes (hospital prestige and professional development opportunities) are positively related to the perceived attractiveness of the hospital as an employer.

Overall, three dimensions (economic, professional and relational attributes) are considered. In addition, the relative importance of these individual attributes and dimensions is determined. Although the importance of an organizational image has received a lot of theoretical attention, relatively few empirical studies have examined this issue. Although research focusing on HPRs has stressed the importance of economic, administrative and professional aspects, these can be as considered isolated studies and little is known about the relative importance in shaping hospital attractiveness.

## Methods

This study was conducted in a medium-sized Belgian academic hospital and concentrates on the medical staff members to study the hospital-physician relationship.

### Instruments

Hospital attractiveness was measured using four items on a seven point Likert scale from 1 = *strongly disagree* to 7 = *strongly agree*, with high scores indicating high attractiveness. This instrument has demonstrated adequate levels of reliability in previous research [33]. Sample items are ‘[hospital name] is attractive to me as a place for employment’ and ‘I would recommend [hospital name] as an employer to my friends’. To measure organizational attributes, the scale from Lievens and colleagues [13] was adapted to the hospital context. By means of focus groups we determined organizational attributes potentially important to predict hospital attractiveness. Two semi-structured interviews with in total sixteen participants were performed. Due to the exploratory nature of our study which concentrated on collecting and testing numerous brief suggestions this number is considered appropriate [34]. Discussion topics were based on the known antecedents of organizational attractiveness and additional antecedents conveyed by the participants. The interviews focused on what employees found important about their job and the organization. This allowed us to drop irrelevant attributes and add relevant ones that were missing. During this process, different antecedents that were identified in previous studies but were not important to the context of the physician-hospital relation were no longer considered (i.e. travel opportunities) while ‘hospital prestige’ was added. During the course of the interview, we increasingly encountered the same organizational characteristics, suggesting that we reached a state of data saturation. The outcomes of the interviews with the focus groups were used to construct a questionnaire. Factor analysis indicated seven factors with an eigenvalue higher than one. As a rule of thumb, items which loaded less than 0.6 on their own factor or more than 0.4 on other factors were removed from the analysis. Therefore, 2 items were omitted resulting in 15 remaining items. The scale items are outlined in Additional file 1. The seven factors correspond with the antecedents that were identified by the focus groups. All items were measured using a 7-point Likert scale.

As a first step, we began checking the internal consistencies of the scales. Internal consistency of the factors was satisfactory, with values for Cronbach’s alpha ranging between 0.69 and 0.98. The instrumental factors are: pay and financial benefits, job security (economic attributes), organizational support, leader support, work-life balance (relational attributes), hospital prestige and professional development opportunities (professional attributes).

A demographic questionnaire was incorporated in the survey to obtain descriptive information. Individuals’ gender, age, tenure within the organization, professional experience, work schedule (full-time versus part-time employment) and whether or not the physician has a leading position were included as covariates in our analyses to rule out potential alternative explanations for our findings. Previous research has shown that these variables are potentially important to understand organizational attractiveness [35].

### Statistical analysis

Data were analyzed using The Statistical Package for Social Sciences (SPSS) version 20.0 for Windows (SPSS, Inc., Chicago, IL, USA). Descriptive statistics, correlations and reliability coefficients were generated for the analyzed variables. We ran a hierarchical regression, controlling for gender, tenure, fulltime versus part-time employment (dummy-coded) and whether or not the respondent has a leading function (dummy coded). Age and professional experience were not used as control variables due to multicollinearity between these two variables and tenure. Because the correlation between these three variables was high (the spearman correlation coefficients were 0.808 and 0.845 respectively), little impact should be expected from omitting both variables.

### Post hoc power test

The data were analyzed by hierarchical multiple linear regression. Because of our limited sample size, a post-hoc sample calculation was performed. Based on a statistical significance level of 0.05, a power of 0.80 and a medium effect size of 0.20, the test revealed that approximately 68 subjects would be needed for a regression analysis with ten independent variables and one dependent variable [36]. Thus, although our sample was relatively small, it had an adequate power to test the stated hypothesis.

### Relative importance analyses

In addition, we examined the relative importance of the organizational attributes in determining organizational attractiveness. However, since the measures of independent variables are interrelated the regression coefficients are not interpretable as measures of relative importance vis-à-vis the others [37] and the regression coefficients were therefore supplemented with relative weights. These relative weights were computed with the analytical approach of Johnson [38]. Relative weights are defined as the proportionate contribution of each independent variable to R^2^, considering both its unique contribution and most importantly also the contribution when combined with other variables. For ease of interpretation we express them as percentages of the predictable variance (R^2^).

### Ethical considerations

Our study was approved by the Medical Ethics Committee of the Université Catholique de Louvain. The questionnaire was distributed to all staff members together with a letter explaining the purpose of the study. Participation to the study was voluntary. Questionnaires were retrieved and processed by non-hospital members to assure anonymity.

## Results

### Participants

The data were collected by paper-and-pencil questionnaires. Although researchers have regularly encountered poor response rates when surveying physicians [39], of the 149 physicians, 86 returned the survey. This represented a satisfactory response rate of 57.8%. This response was felt to be adequate for an exploratory study of the instrument to the HPR-setting. Sample characteristics are included in Table 1. Most participants were male (54.7%) and were fulltime employed (76.7%). The physicians were on average 45 years old and had more than 10 years experience in the organization (53.6%). These figures are comparable with the characteristics of the whole medical staff (66% are male, 88% are fulltime employed and had on average 10.7 years experience in the organization).

**Table 1:** Participants’ demographics

	**N**	**%**
Gender		
Male	47	54.7
Female	39	45.3
Age		
Ranges from 26 to 64	Mean = 44.88	SD = 10.32
Organizational tenure		
< 5 years	25	29.8
5 to 10 years experience	14	16.6
10 to 20 years experience	26	31.0
> 20 years experience	19	22.6
Fulltime employment	66	76.7
Leading position	24	27.9

### Descriptive statistics

Table 2 presents the means, standard deviations and correlations of these variables in this study. Internal consistencies are on the diagonal. All variables were significantly related to hospital attractiveness. This is not surprising in light of our qualitative pre-study to identify relevant variables. To test our hypotheses we conducted a multiple regression analysis.

**Table 2:** Means, standard deviations and correlations of study variables

	**Mean**	**SD**	**1.**	**2.**	**3.**	**4.**	**5.**	**6.**	**7.**	**8.**	**9.**	**10.**	**11.**	**12.**
Personal characteristics														
1. Gender	0.55	0.50	-											
2. Tenure	44.88	10.32	0.291**	-										
3. Employment status	12.99	9.67	0.370**	0.200	-									
4. Leading position	0.78	0.42	0.191	0.391**	0.197	-								
Hospital characteristics														
*Economic attributes*														
5. Pay and financial benefits	4.09	1.41	0.003	0.073	−0.09	−0.002	*0.945*							
6. Job security	5.21	1.29	−0.012	0.066	0.091	−0.118	0.14	*0.703*						
*Relational attributes*														
7. Organizational support	5.35	1.11	−0.063	0.05	−0.057	0.269*	0.371**	0.165	*0.903*					
8. Leader support	3.15	1.60	−0.126	−0.272*	−0.238*	−0.19	0.194	0.286**	0.294**	*0.981*				
9. Work-life balance	4.86	1.72	−0.174	−0.298**	−0.263*	−0.279*	0.407**	0.195	0.452**	0.426**	*0.793*			
*Professional bttributes*														
10. Hospital prestige	3.57	1.46	0.206	−0,066	0.135	0.033	0.184	0.316**	0.455**	0.237*	0.259*	*0.689*		
11. Professional development opportunities	5.11	1.19	−0.115	−0.115	−0.108	0.05	0.216*	0.327**	0.547**	0.528**	0.418**	0.444**	*0.702*	
Dependent variable														
12. Hospital attractiveness	5.29	1.08	0.037	0.053	−0.04	0.015	0.408**	0.300**	0.606**	0.529**	0.389**	0.588**	0.702**	*0.918*

*Correlation is significant at the 0.05 level (2-tailed).

**Correlation is significant at the 0.01 level (2-tailed).

### Impact of hospital attributes

Based on hierarchical linear regression analysis, the set of hospital attributes was found to have a significant and positive effect on organizational attractiveness. The attributes jointly explained a significant amount of variance (Adjusted R^2^ = 0.793; P < 0.001). This high amount can be explained by the holistic view we applied to the HPR and the thorough build-up of our model by means of a literature review and focus groups. Table 3 provides an overview.

**Table 3:** Regression analysis

	**Relative weights**	**Relative weights as % of R**^**2**^	**Relative weights**	**Relative weights as % of R**^**2**^
Personal characteristics			3.3%	4.1%
Gender	0.6%	0.75%		
Tenure	0.8%	1.06%		
Fulltime employment	0.2%	0.31%		
Leading	1.6%	2.02%		
Organizational attributes			76.0%	95.9%
*Economic attributes*			*10.9%*	
Pay and financial benefits	7.4%	9.28%		
Job security	3.6%	4.51%		
*Relational attributes*			*29.7%*	
Organizational support	17.2%	21.66%		
Leader support	9.3%	11.67%		
Work-life balance	3.3%	4.14%		
*Professional attributes*			*35.3%*	
Hospital prestige	16.5%	20.84%		
Professional development opportunities	18.8%	23.75%		
R^2^	0.793			

In the first step, the control variables were added. Having a leading position within the hospital (P = 0.002; β = -0.248) and tenure (P = 0.046; β = 0.148) were significant predictors of hospital attractiveness. The explained variance was however limited (for leadership 0.8% and tenure 1.6%). Gender (P = 0.900; β = -0.009) and full-time employment (P = 0.477 β = -0.048) were no statistically significant predictors.

In the second step, the organizational attributes were added. Our organizational attributes explained 76.0% of the variance. Professional attributes were identified as the strongest predictors (35.3%); professional development opportunities (P = 0.003, β = 0.280) explained 18.8 % of the variance and hospital prestige (P < 0.001, β = 0.291) explained 16.5%. This confirmed the argument noted by the participants of the exploratory focus groups which led to the inclusion of prestige as an additional hospital characteristic. Besides professional aspects of the HPR, relational attributes were found to be important (29.7%). Organizational support (P = 0.001; β = 0.337) explained 17.2% variance; leader support (P = 0.033; β = 0.170) explained 9.3% variance and work-life balance (P = 0.156; β = -0.125) 3.3%. Third, economic aspects accounted for 10.9% of variance. Pay and financial benefits (P = 0.004; β = 0.203) explained 7.4% and job security 3.6% (P = 0.642; β = 0.033). The economic attributes are less important than the non-economic attributes (relational and professional attributes) mentioned above. Table 3 provides a full overview. The first two columns present the relative weights and the percentage of predictable variance (relative weights as a percentage of R^2^). The last two columns provide an overview of the aggregated relative weights and percentage of predictable variance of the personal characteristics, economic, relational and professional attributes.

## Discussion

A key aim of this study was to address the lack of research on the relative importance of different hospital attributes that determine hospital attractiveness to physicians. In light of the physician shortage [4], the physician fled to self-owned ambulatory facilities [2] and increased competition between general hospitals [12], the insights developed by this study are of major importance.

First, our findings demonstrate the importance of professional attributes. Both hospital prestige and opportunities for physicians to develop themselves professionally were major predictors of hospital attractiveness. These findings confirm the results of previous research [19,32] that showed that the psychological contract of physicians also consists of a professional dimension. Therefore it is clear that the broader institutional context of the HPR cannot be neglected. However, the professional aspects of the HPR remain largely an unexplored terrain. While we increase insight by exploring the importance physicians’ perceptions of hospital prestige and professional development opportunities future research needs to clarify this issue further.

Second, relational attributes of hospitals were also identified as an important predictor of hospital attractiveness. This finding is supported by the rich theoretical and empirical evidence rooted within social exchange. At the core of this approach is the norm of reciprocity which is described as the social expectation that people respond positive to positive actions [40]. More precisely, perceived organizational and leader support have been identified as strong predictors of a wide variety of organizationally desired work attitudes and behaviour (e.g. job satisfaction, organizational trust, organizational citizenship behaviour) [30,41]. We contribute to the body of knowledge by demonstrating the significance of both organizational and leader support to organizational attractiveness. Furthermore, we showed that work-life balance did not predict hospital attractiveness. This is surprising since the business case for work-life balance practices relies on the ability to reduce work-life conflict thereby potentially improving employee attitudes and behaviors within the organization [31]. However this result could be interpreted in light of the importance of professional attributes mentioned above. Professional development and prestige contrasts to a certain extent the desire to preserve leisure and family time. However, this needs further clarification. Moreover since healthcare workers experience frequently high levels of work-related stress and burn-out [42] accentuating the importance of healthy well-being at work we argue that the importance of work-life balance to professionals is an interesting direction for future research.

Third, our findings confirm the statement that the economic relationship between hospital and physician is only of limited importance. This contrasts the focus of previous research which has concentrated predominately on financial alignment issues between both parties [17]. Moreover, these studies assume that human motivation is primarily based on self-interest and ignore the fact that economic transactions are embedded in social relationships. Our finding highlights the fact that physicians, as professionals, have a more complex set of motives that underlie their behavior. This confirms Herzberg’s [43] view on financial conditions which in the two factor theory are identified as a hygiene factor which does not give positive satisfaction, though dissatisfaction results from its absence. Furthermore, hospitals are practicing in an increasingly competitive environment characterized by a physician shortage in which financial conditions cannot be neglected. However, in general we advise hospital administrators and policy makers not to reduce the HPR to a financial or economic relationship and apply a diverse strategy in which besides economic ties, also relational and professional aspects are considered.

However, in light of this finding it is important to note that this study focused on employed physicians practicing at a university hospital and it could be that this issue is of greater importance to a setting in which physicians are self-employed. This issue warrants further research.

Finally, our quantitative study did not identify job security as an important predictor. Bearing in mind that the physician labor market is characterized by a chronic physician shortage this finding is not that surprising. However, this confirms and highlights the importance of hospital management to increase hospital attractiveness in order to retain scarce physicians in a highly competitive labor market.

### Limitations

The cross-sectional nature of our study precludes strong claims of causality. A longitudinal study to examine changes over time would be valuable. Furthermore, our study comprises a small sample size and includes only one Belgian academic hospital. It would be insightful to replicate this study using a larger representative sample of hospitals. In addition, it would be valuable to perform an international study that also considers differences between different types of health care systems and countries. However, the theoretical support for our results and findings of previous research with potential applicants and employees outside the healthcare setting is encouraging and suggests that further research is warranted. More specifically, since operational linkages with the hospital (i.e. the use of the operating theatre and supporting personnel) and remuneration (i.e. medical fees) differs between medical specialties, a study focusing on the potential differences of attributes between different types of physicians (e.g. pediatrics vs. orthopedics) would be interesting. Also, our study focused on a large academic hospital. It would be valuable to study differences between physicians practicing at academic hospitals and physicians practicing at general hospitals. Moreover the opportunities with respect to teaching, research and opportunities to deliver highly (sub)specialized care differ between academic and non-academic hospitals and therefore the relative importance of hospital attributes could be different. In addition, it is important to note that in Belgian academic hospitals physicians are salaried employees. This contrast with the setting of self-employed physicians. It is likely that the different economic ties shape the hospital-physician relationship to a great extent. Moreover, the difference in the relative importance of economic and the various non-economic factors to self-employed physicians would be interesting to investigate. Studies focusing on these other settings provide valuable avenues for future research. Finally, the impact of hospital attributes and attractiveness to physicians on other important managerial outcomes such as retention of physicians, organizational attitudes (e.g. organizational commitment) and performance (e.g. organizational citizenship behavior) pose interesting possibilities for future research.

## Conclusions

In this study we conceptualized hospital attractiveness to physician specialists as a package of organizational attributes. We examined the relative importance of these attributes in shaping the organizational image thereby determining organizational attractiveness to physicians practicing at that hospital. Our results show that hospital attractiveness is primarily determined by non-economic factors. Hospital attractiveness is most strongly predicted by the professional attributes (professional development opportunities and prestige). Furthermore relational attributes are important (organizational support and leader support). Work-life balance and job security did not contribute significantly. In addition, physicians indicated pay and financial benefits as an economic predictor of hospital attractiveness. However, this economic dimension of the hospital-physician relationship is less important than the non-economic characteristics contributing to an attractive work environment.

## Competing interests

The authors declare that they have no competing interests.

## Authors’ contributions

All authors made substantial contributions to the conception and design, acquisition of data, or analysis and interpretation of the data. All authors were involved in drafting the manuscript and revising it for important intellectual content and gave final approval of the version to be published.

## Pre-publication history

The pre-publication history for this paper can be accessed here:

http://www.biomedcentral.com/1472-6963/14/232/prepub

## Supplementary Material

Additional file 1 Scale items for hospital attributes (7-point Likert scale). Click here for file
