# Supplementary material for: Hospital-physician relations: the relative importance of economic, relational and professional attributes to organizational attractiveness
Source: BMC Health Serv Res. 2014 May 21;14:232. doi: 10.1186/1472-6963-14-232 (PMC4055796; doi:10.1186/1472-6963-14-232)
Supplement: Additional file 1 — Scale items for hospital attributes (7-point Likert scale). [file 1472-6963-14-232-S1.docx]

Additional file 1

Scale items for hospital attributes (7-point Likert scale)

| Organizational Attributes | |
| --- | --- |
| *Economic Attributes* |  |
| Pay and Financial Benefits | I am rewarded fairly for the effort that I put in my work. |
|  | I am rewarded fairly considering the responsibilities that I have. |
| Job Security | [hospital name] offers me job security. |
|  | [hospital name] offers me a job for life. |
| *Relational Attributes* |  |
| Organizational Support | [hospital name] listen to its employees. |
|  | [hospital name] know how to value its employees. |
| Leader Support | My immediate supervisor can be relied upon when things get tough on my job. |
|  |  |
|  | My immediate supervisor is willing to listen to my job-related problems. |
| Work-Life Balance | [hospital name] offers me a job with good working hours. |
|  | [hospital name] makes efforts to diminish work pressure. |
|  | [hospital name] makes efforts to provide a good work-life balance. |
| *Professional Attributes* |  |
| Hospital Prestige | [hospital name] is well respected in [region name]. |
|  | The academic status of [hospital name] is very appealing to me. |
| Professional Development Opportunties | [hospital name] offers me the opportunity to build a career |
|  | hospital name] offers me enough opportunities for training and education |
|  |  |
| Hospital Attractiveness | |
|  | [hospital name] is attractive to me as a place for employment. |
|  | I would recommend [hospital name] as an employer to my friends. |
|  | You would advise potential candidates to apply with [hospital name]. |
|  | For me, [hospital name] is an attractive employer. |
